# Microbial risks in drinking water systems: persistence and public health implications of opportunistic premise plumbing pathogens

**DOI:** 10.3389/fmicb.2025.1575789

**Published:** 2025-05-08

**Authors:** Claire Hayward, Kirstin E. Ross, Melissa H. Brown, Richard Bentham, Muhammad Atif Nisar, Jason Hinds, James Xi, Harriet Whiley

**Affiliations:** ^1^Future Industries Institute, University of South Australia, Mawson Lakes, SA, Australia; ^2^Environmental Health, College of Science and Engineering, Flinders University, Bedford Park, SA, Australia; ^3^College of Science and Engineering, Flinders University, Bedford Park, SA, Australia; ^4^ARC Training Centre for Biofilm Research and Innovation, Flinders University, Bedford Park, SA, Australia; ^5^Built Water Solutions, Clarendon, SA, Australia; ^6^Enware Australia Pty Ltd., Caringbah, NSW, Australia

**Keywords:** opportunistic premise plumbing pathogens, biofilms, free living amoeba, drinking water, healthcare associated infections

## Abstract

**Introduction:**

The persistence of opportunistic premise plumbing pathogens (OPPPs) in drinking water plumbing systems poses a significant public health risk that is receiving increasing attention yet remains poorly understood. This study investigated the co-occurrence of OPPPs and the influence of building infrastructure properties on their prevalence.

**Methods:**

Drinking water and biofilm samples were collected from hospitals and private residences across Australia to investigate the abiotic and biotic factors contributing to the growth and proliferation of OPPPs.

**Results:**

Quantitative polymerase chain reaction assays revealed that 41% of samples tested positive for *Pseudomonas aeruginosa*, 26% for *Staphylococcus aureus*, 26% for *Legionella* spp., 24% for *Legionella pneumophila*, and 14% for *Acinetobacter baumannii*. Furthermore, free-living amoebae, including *Vermamoeba vermiformis* (46%) and *Acanthamoeba* spp. (25%), were frequently detected, with *Acanthamoeba* spp. demonstrating a significant positive correlation with all bacterial OPPPs. Overall, results indicated a statistically higher prevalence of OPPPs in residential properties and in biofilms. However, building characteristics, including stagnation, hot water system type, and building age, had inconsistent influences on individual OPPP prevalence.

**Discussion:**

These results emphasize the need to incorporate risk assessments regarding the complex factors within the premise plumbing environment that contribute to pathogen persistence, to inform evidence based targeted preventative strategies for at-risk populations. These findings are particularly critical for individuals receiving healthcare at home, as inconsistent water treatment and monitoring in residential settings may increase their risk of exposure to OPPPs.

## 1 Introduction

Opportunistic premise plumbing pathogens (OPPPs), such as *Legionella pneumophila, Pseudomonas aeruginosa* and *Acinetobacter baumannii* can persist in drinking water plumbing environments (Falkinham et al., 2015). These pathogens are capable of surviving under low-nutrient conditions, in protozoan hosts and in biofilms formed on the surface of plumbing systems (Hayward et al., 2022a). The presence of OPPPs in drinking water plumbing in receiving increasing attention, particularly in the healthcare space (Perkins et al., 2019). The incidence of OPPP related infection has overtaken enteric pathogen infections as the leading cause of water related outbreaks (Beer et al., 2015; Collier et al., 2021). It is estimated that approximately 7.15 million waterborne illnesses occur each year in the United States (US), and most of hospitalisations and deaths were caused by biofilm-associated pathogens including *Pseudomonas* spp., *Legionella* spp. and nontuberculous mycobacteria (Collier et al., 2021). The significance of OPPPs is exacerbated by antimicrobial resistance and virulence factors (LeChevallier et al., 2024; Hayward et al., 2022a). The burden of disease from OPPPs such as *P. aeruginosa* and *A. baumannii* is unclear as these infections are rarely nationally notifiable (Commonwealth of Australia, 2024, Centers for Disease Control and Prevention, 2024). Furthermore, the estimates of OPPP HAIs are an underestimation as the incidence of community acquired infection remains unclear (Collier et al., 2021; Hayward et al., 2022b).

Traditionally, drinking water plumbing systems were considered a low risk for diverse microbial communities due to the harsh environmental conditions (LeChevallier et al., 2024). However, recent literature has demonstrated that clinically relevant pathogens can survive in drinking water plumbing systems, and that point of use water related devices may be contaminated by the user (Nisar et al., 2023; Kelly et al., 2014; Huang et al., 2021; Hayward et al., 2024). Previous research has found 44% of water and biofilm samples positive for *Legionella* spp. and 55% for *Vermamoeba vermiformis* gene markers (Nisar et al., 2022). These pathogens may be transmitted via consumption, inhalation or from contact with a contaminated water source (Dean and Mitchell, 2020; World Health Organization, 2016). Previous research has focussed on the aerosolization of *Legionella* spp. from showers and tap faucets (Kanamori et al., 2016; Chang and Hung, 2012; Bollin et al., 1985). However, it is unclear if the other OPPPs are also primarily transmitted via the same pathways, considering the diverse range of potential infection types including wound, catheter and central line infections (Qin et al., 2022; Ayoub Moubareck and Hammoudi Halat, 2020; Tong et al., 2015).

Completely preventing the colonization of drinking water plumbing systems with OPPPs is unrealistic. It is impractical for water utilities to implement major changes in water temperature or residual disinfection throughout an entire drinking water distribution system. Particularly when these pathogens can be protected from traditional treatment methods by residing in biofilms (Falkinham, 2015). This is further complicated by their survival in free living amoeba hosts, a role that is not well understood for many clinically relevant bacteria (Nisar et al., 2022; Isaac and Sherchan, 2020; Gomez-Alvarez et al., 2023; Shaheen et al., 2019). Consequently, effective management of building water systems is crucial to control the risk posed by the growth and proliferation of these pathogens at the point of use. The prevalence of OPPPs in building water systems is influenced by a number of factors such as building size, plumbing system age, flow rate, temperature and water storage (Leslie et al., 2021; Dai et al., 2018; Brazeau and Edwards, 2011; Nisar et al., 2023). Buildings without evidence-based water management protocols can support OPPP growth and proliferation in drinking water by increasing stagnation, inadequate temperature control and reduction in disinfectant residual (Ley et al., 2020; Rhoads et al., 2016). Therefore, a multi-barrier approach is required to control the growth and proliferation of these functionally diverse pathogens (Hayward et al., 2022b; Leslie et al., 2021). Before an appropriate combination of barriers can be determined, it is important to know what factors, both biotic and abiotic, contribute to the persistence of these pathogens in complex drinking water plumbing systems.

There are limited studies comprehensively investigating the presence of OPPPs in Australia drinking water (Nisar et al., 2022; Höllhumer et al., 2020; Puzon et al., 2020; Ahmed et al., 2017; Kaestli et al., 2019; Marshall et al., 2011). More broadly, there are limited studies investigating the relationships between OPPPs and their protozoan hosts (Shanan et al., 2015; Valster Rinske et al., 2011; Nisar et al., 2022). In the present study, water and biofilm samples were collected from hospital and residential building drinking water plumbing systems and were screened for the presence of *Legionella, L. pneumophila, P. aeruginosa, A. baumannii, S. aureus, Acanthamoeba* and *V. vermiformis*. This is the first comprehensive study which used molecular tools for the screening of multiple OPPPs in Australian domestic and hospital drinking water plumbing systems, offering actionable recommendations for developing more effective water management protocols and public health strategies to mitigate the risk of pathogen exposure in healthcare and residential settings. These findings are particularly relevant for shaping policy and intervention strategies focused on improving water quality management in buildings with at-risk populations.

## 2 Methods

### 2.1 Sample collection and processing

This study was approved by the Flinders University Social and Behavioral Research Ethics Committee (SBREC Project Number 7291). From February 2019 to May 2024, 218 water and 182 biofilm samples were collected from showers, faucets, drains, baths, basins and overflows from domestic and healthcare facility water systems. There were 154 domestic samples and 246 hospital samples collected across New South Wales and South Australia. Residential settings were included to provide a comparative analysis of pathogen prevalence between healthcare and non-healthcare environments. Due to ethical policies, the authors cannot disclose the geographic location of these premises. The physical and environmental parameters of the sampling site were recorded upon sampling where possible including outlet usage frequency, water source, building age, plumbing system age, water heating system and hot water storage (Supplementary Tables S2, S3). Water and biofilm samples were transported according to the Centers for Disease Control and Prevention guidelines (Centers for Disease Control and Prevention, 2019b). Briefly, 1 L potable water samples were collected in sterile screw capped wide mouth plastic bottles (2105–0032 Nalgene) containing 1 mL 0.1 M Na_2_S_2_O_3_ (124,270,010, ACROS Organics™) to neutralize residual chlorine-based disinfectants. Sterile polyurethane-tipped swabs (CleanFoam®TX751B, Texwipe®) were used to collect biofilms. These swabs were moistened with sterile water and the surface of the faucet aerator or drain was swabbed for 10 s. The swab was then placed in a 10 mL screw capped vial with 5 mL of 1X sterile phosphate buffered saline (PBS). All samples were stored at 5°C and analyzed within 72 h of collection. All water and biofilm samples were vacuum filtered onto a 47 mm diameter 0.2 μm polycarbonate membrane (GTTP04700, Isopore™). The membrane was then transferred to a sterile 10 mL screw top vial containing 3 mL of sterile PBS followed by 5 min shaking (Griffin Flask Shaker), vortexing (SEM® Vor Mix) and sonication (CooperVision® 895 Ultrasonic Cleaner). This suspension was used for molecular analysis.

### 2.2 Microbial testing

DNA was extracted for quantitative polymerase chain reaction (qPCR) analysis from 1 mL of the concentrated water sample or resuspended biofilm sample using the BIO-RAD.

Aquadien™ DNA extraction and purification kit following the manufacturer’s instructions.

(Bio-Rad Laboratories, Inc., Sydney, NSW, Australia). Ten μL lysozyme (89833) (ThermoFisher Scientific: Adelaide, Australia) (25 mg/mL in 1X PBS) was added to the extraction sample and incubated at 37°C for 15 min prior to the boiling step.

The ISO/TS12869:2019 standard qPCR assay was used to enumerate the 16S rDNA *Legionella* spp. gene and *L. pneumophila mip* gene (International Organization for Standardization, 2019). The 18S rDNA gene was amplified to quantify *Acanthamoeba* and *V. vermiformis* (Qvarnstrom et al., 2006; Scheikl et al., 2016). The *ompA*, *gyrB* and *nuc* genes were used to quantify *A. baumannii, P. aeruginosa* and *S. aureus,* respectively (McConnell Michael et al., 2012; Lee et al., 2011; Galia et al., 2019)*. Legionella* spp.(GenBank Acc CP021281), *L. pneumophila* (GenBank Acc KR902705), *Acanthamoeba castellanii* (GenBank Acc U07413), *V. vermiformis* (GenBank Acc KT185625), *A. baumannii* (GenBank Acc OL347635.1), *P. aeruginosa* (GenBank Acc HQ425720.1) and *S. aureus* (GenBank Acc GQ370471.1) gBlock gene fragments (IDT™) were used to create a standard curve using 10fold serial dilutions. The qPCR efficiency and limit of detection for each assay was validated by generating standard curves from these serial dilutions, where the efficiency was calculated based on the slope of the curve and was found to be within the acceptable range. qPCR reaction mixes consisted of specific oligos (BIO-RAD Laboratories Ltd.), 2X Sso.

Advanced™ universal probe supermix (172–5,281, BIO-RAD Laboratories Ltd.) and template DNA were used in a Rotor-Gene Q thermal cycler (QIAGEN Ltd.). Sequences of oligos and qPCR conditions are described in Supplementary Table S1. All assays were performed in triplicate and mean Ct values were used for estimation of the genomic unit litre of water (GU/L) and genomic unit per mL of biofilm (GU/swab).

### 2.3 Statistical analysis

Data analyses were performed using SPSS and R software. Descriptive statistics were first computed to summarize the data, followed by correlation analyses using Spearman’s rank correlation, as the data were not normally distributed. The results were interpreted at the level of significance *p* < 0.05, with a 95% confidence interval for all statistical tests. Exact significance values are reported in the results section.

## 3 Results

### 3.1 Abiotic factors

Abiotic factors including type of water heating system, hot water storage, building age, plumbing system age and sampled outlet usage frequency were recorded for all domestic water system samples.

Overall, 52% (*n* = 80) of samples were collected from residential properties with gas hot water heating systems, 25% (*n* = 39) had electric systems, 1.3% (*n* = 2) had solar systems and 21% (*n* = 33) of respondents did not know what system their property had. Half (50%, *n* = 78) of samples were collected from properties that did not have hot water storage (instantaneous hot water heating), 19% (*n* = 29) did have hot water storage and 31% (*n* = 47) of samples were collected from properties where the resident did not know if they had hot water storage or not.

Overall, 64% (*n* = 99) of samples were collected from properties more than 20 years old, 12% (*n* = 19) were from buildings less than 5 years old, 5.8% (*n* = 9) were taken from buildings that were 5–9 years old and 18% (*n* = 27) were collected from buildings where the resident could not estimate how old the building was. Regarding plumbing system age, 28% (*n* = 43) of samples were collected from outlets that were less than 5 years old, 25% (*n* = 38) were collected from outlets that were more than 20 years old, 19% (*n* = 29) were collected from outlets that were 5–9 years old, 10% (*n* = 16) from outlets 10–14 years old and 18% (*n* = 28) were collected from outlets where the residents did not know how old it was.

Most samples, 61% (*n* = 94), were collected from outlets used 2–10 times per day. 15% of samples were collected from outlets used more than 10 times per day, 5.2% (*n* = 8) from outlets used less than once per month, 1.9% (*n* = 3) from outlets used once per week, 1.3% (*n* = 2) from outlets used once per fortnight, and, 15% (*n* = 24) were collected from outlets where the resident did not know how frequently it was used.

### 3.2 Amoeba

#### 3.2.1 Vermamoeba vermiformis

Overall, 46% (*n* = 183/400) of total (residential and hospital) samples were positive for *V. vermiformis* (18S rDNA gene) with a concentration range of 2.7 × 10^2^ to 7.47 × 10^7^ GU/L and 1.2 × 10^2^ to 3.45 × 10^8^ GU/swab (Supplementary Table S3). There was no statistically significant difference in *V. vermiformis* prevalence between residential and hospital buildings (*p* = 0.261) (Table 1). Furthermore, there was no statistically significant difference in prevalence between water or biofilm samples (*p* = 0.197) or between outlet types (*p* = 0.065) (Table 1).

**Table 1:** Prevalence of target opportunistic premise plumbing pathogens in residential and hospital water systems detected by 214 quantitative polymerase chain reaction.

	*Vermamoeba vermiformis* (18S rDNA gene)		*Acanthamoeba* spp. (18S rDNA gene)		*Pseudomonas aeruginosa* (*gyrB* gene)		*Staphylococcu s aureus* (*nuc* gene)		*Legionella* spp. (16S rDNA gene)		*Legionella pneumophila* (*mip* gene)		*Acinetobacter baumannii* (*ompA* gene)	
**Water**														
Domesti c	38.98% (*n* = 23/59)	***P* = 0.932**	11.86% (*n* = 7/59)	***P* = 1**	45.09% (*n* = 23/51)	***P* = 0.394**	27.08% (*n* = 13/48)	***P* = 1**	59.32% (*n* = 35/59)	***P* = 0.001**	40.67% (*n* = 24/59)	***P* = 0.3**	2.12% (*n* = 1/47)	***P* = 0.825**
Hospital	49.36% (*n* = 78/158)		14.46% (*n* = 23/159)		33.02% (*n* = 36/109)		24.13% (*n* = 21/87)		27.04% (*n* = 43/159)		23.27% (*n* = 37/159)		3.7% (*n* = 4/108)	
**Total**	**46.54%** **(*n* = 101/217)**		**13.76% (*n* = 30/218)**		**36.88% (*n* = 59/160)**		**25.19% (*n* = 34/135)**		**35.77% (*n* = 78/218)**		**27.98% (*n* = 61/218)**		**3.23% (*n* = 5/155)**	
**Biofilm**														
Domestic	46.31% (*n* = 44/95)	***P* = 1**	58.94% (*n* = 56/95)	***P* = 0.001**	63.04% (*n* = 58/92)	***P* = 0.001**	34.06% (*n* = 31/91)	***P* = 0.101**	36.31% (*n* = 25/95)	***P* = 0.199**	18.94% (*n* = 18/95)	***P* = 0.219**	34.06% (*n* = 31/91)	***P* = 0.002**
Hospital	43.67% (38/87)		14.94% (*n* = 13/87)		12.76% (*n* = 6/47)		12.76% (*n* = 6/47)		11.49% (*n* = 10/87)		8.04% (*n* = 7/87)		12.76% (*n* = 6/47)	
**Total**	**45.05% (*n* = 82/182)**		**37.91% (*n* = 69/182)**		**46.04% (*n* = 64/139)**		**26.81% (*n* = 37/138)**		**19.23% (*n* = 35/182)**		**13.73% (*n* = 25/182)**		**26.81% (*n* = 37/138)**	

Statistically significant relationships defined as *p* < 0.05 and denoted in green. Relationships not statistically significant (*p* > 0.05) denoted in red.

**Figure 1:**
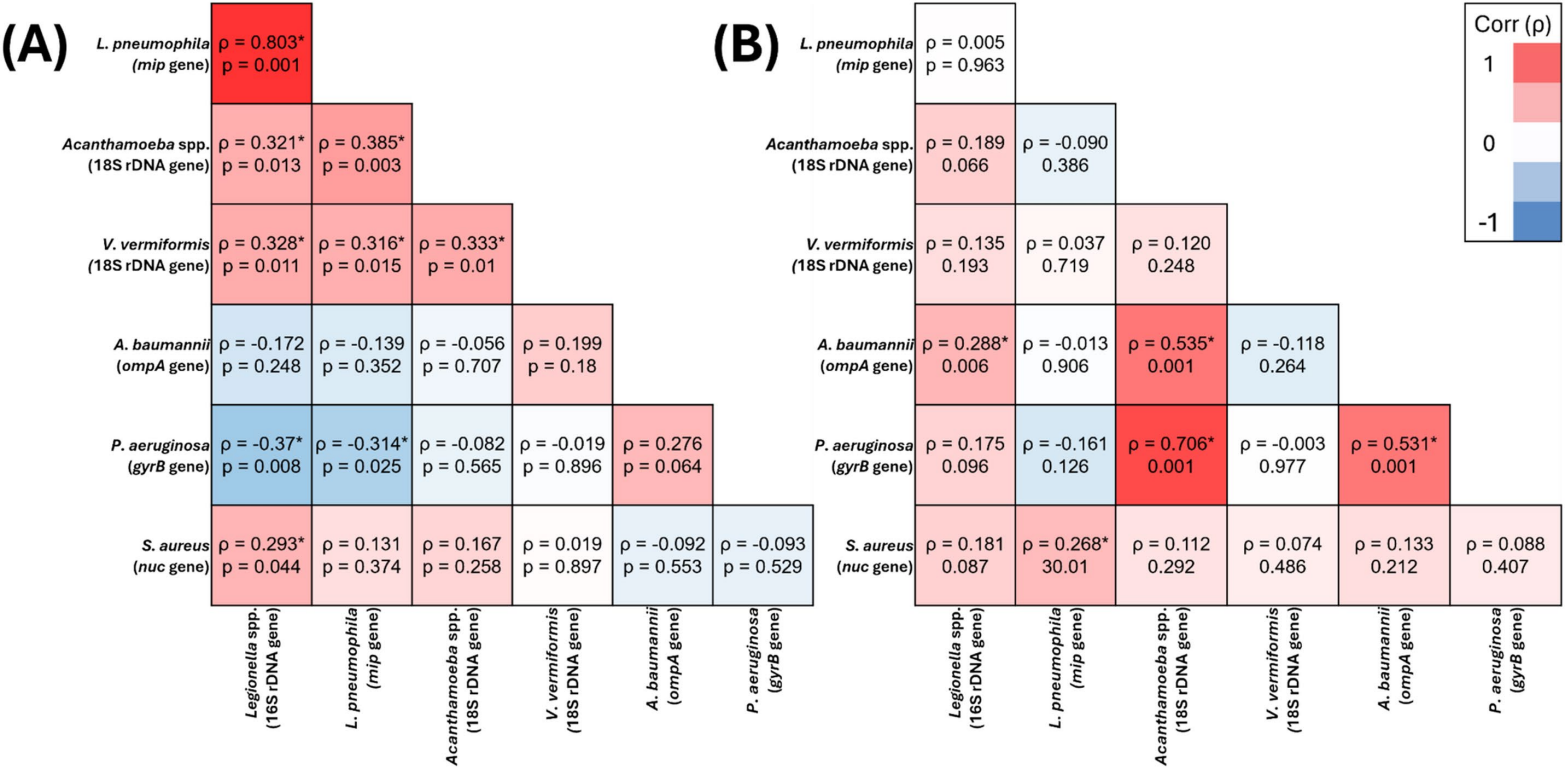
Spearman’s correlation analysis of the presence of target opportunistic premise plumbing pathogens in residential water **(A)** (*n* = 59) and residential biofilm **(B)** (*n* = 95) samples The heat map values show the Spearman’s correlation coefficient (*ρ*) to a significance threshold of *p* < 0.05 (* indicates significant relationships), ranging from −1.0 (blue) to 1.0 (red). A minus value demonstrates a negative association, whereas a positive value demonstrates positive association.

**Figure 2:**
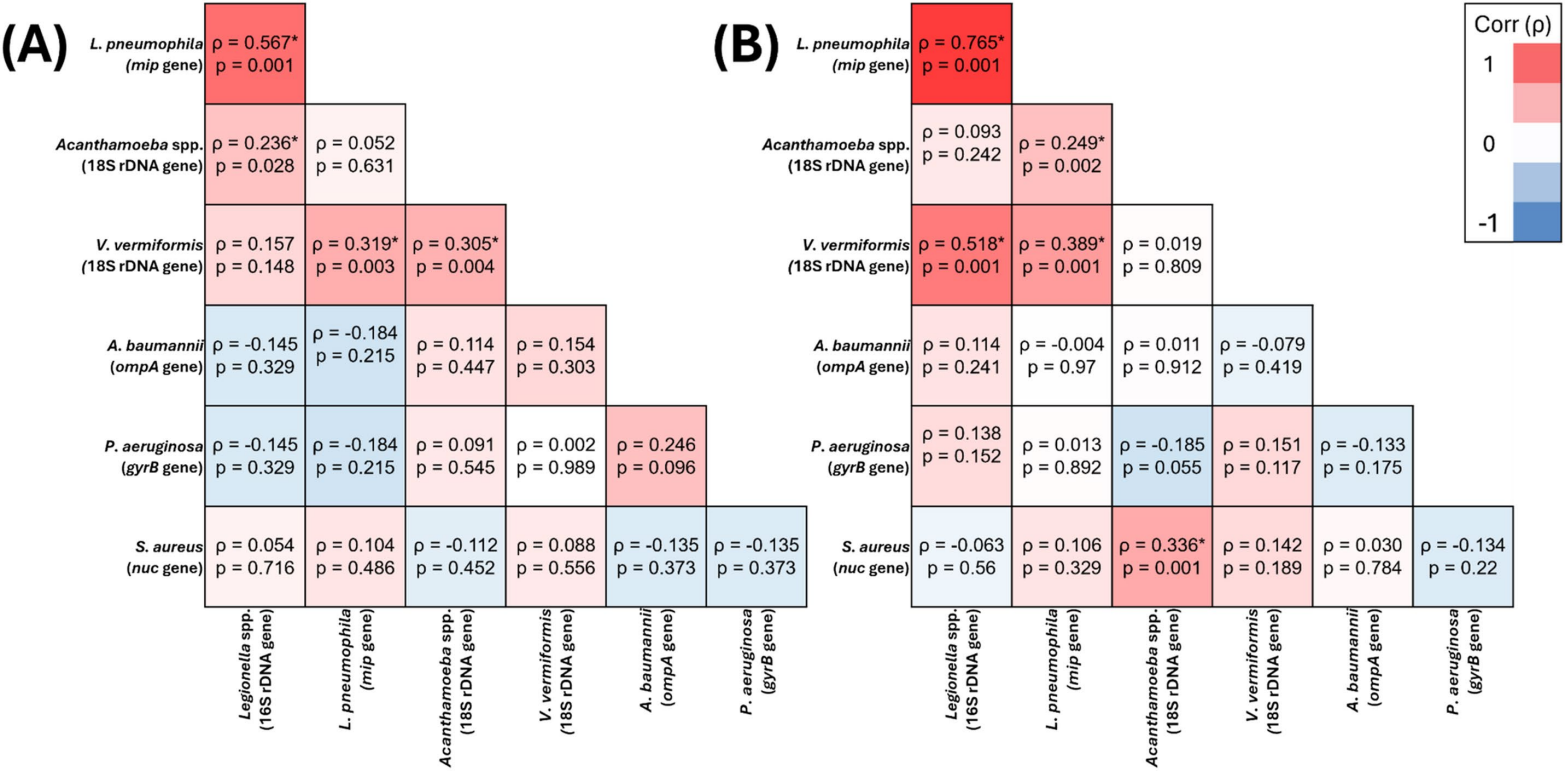
Spearman’s correlation analysis of the presence of target opportunistic premise plumbing pathogens in hospital water **(A)** (*n* = 159) and hospital biofilm **(B)** (*n* = 87) samples The heat map values show the Spearman’s correlation coefficient (*ρ*) to a significance threshold of *p* < 0.05 (* indicates significant relationships), ranging from −1.0 (blue) to 1.0 (red). A minus value demonstrates a negative association, whereas a positive value demonstrates positive association.

In residential water, *V. vermiformis* was significantly positively correlated with *Legionella* spp. (*ρ* = 0.328, *p* = 0.011), *L. pneumophila* (*ρ* = 0.316, *p* = 0.015) and *Acanthamoeba* spp. (*ρ* = 0.333, *p* = 0.01) (Figure 1A), however, its presence it was not significantly correlated with any of the target OPPPs in residential biofilms (Figure 1B). In hospital water, *V. vermiformis* was significantly positively correlated with *L. pneumophila* (*ρ* = 0.389, *p* = 0.001) (Figure 2A), and in biofilms it was significantly positively correlated with *L. pneumophila* (*ρ* = 0.319, *p* = 0.003) and *Acanthamoeba* spp. (*ρ* = 0.305, *p* = 0.004) (Figure 2B).

**Figure 3:**
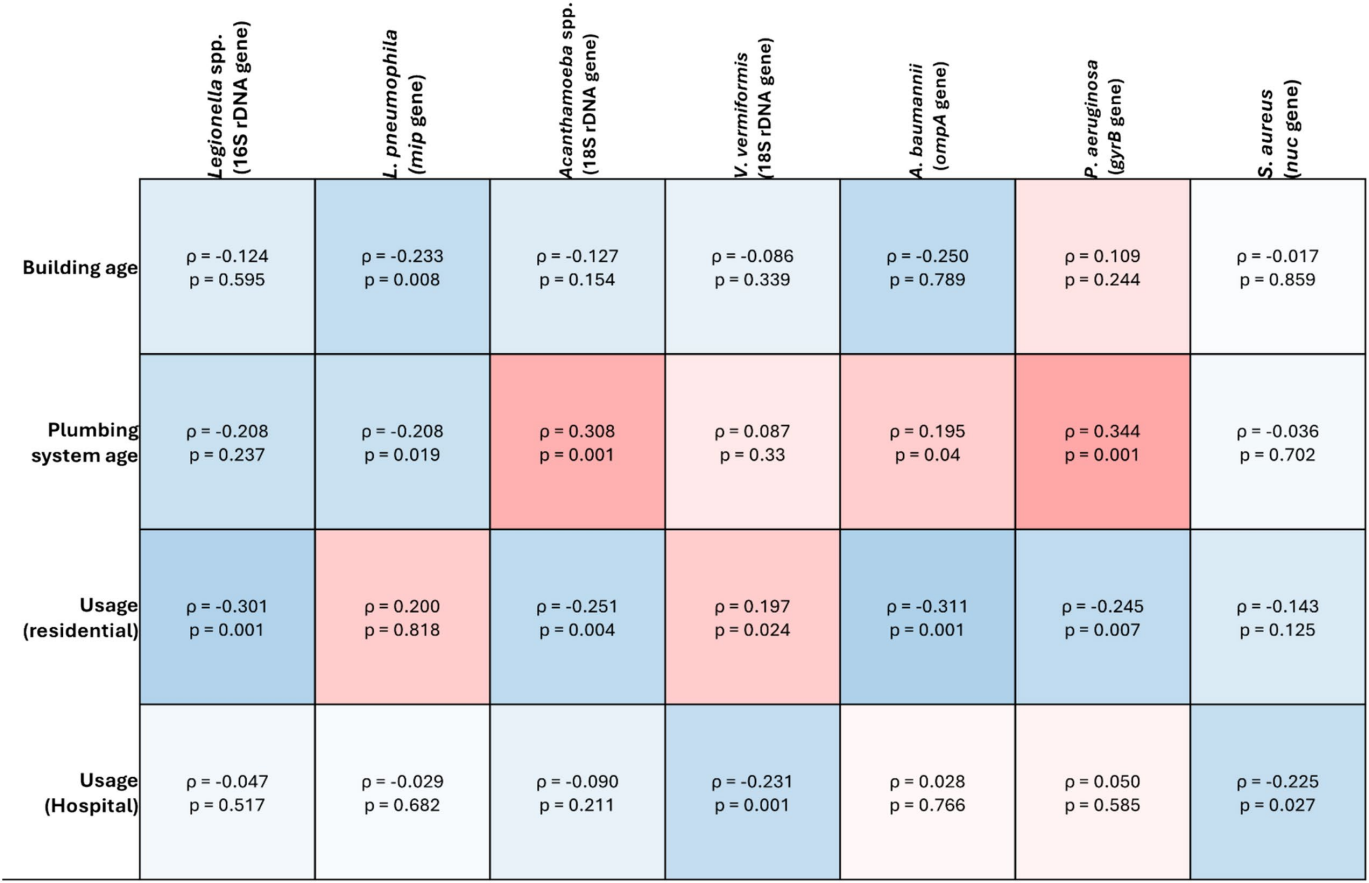
Spearman’s correlation analysis of the target opportunistic premise plumbing pathogens against abiotic factors in drinking water plumbing systems. The heat map value shows the Spearman’s correlation coefficient (*ρ*) to a significance threshold of *p* < 0.05, ranging from −1.0 (blue) to 1.0 (red). A minus value demonstrates a negative association, whereas a positive value demonstrates positive association.

Prevalence of *V. vermiformis* increased as outlet usage decreased in both hospitals (*ρ* = −0.231, *p* = 0.001) and residential buildings (−*ρ* = 0.197, *p* = 0.024) (Figure 3).

#### 3.2.2 *Acanthamoeba* spp.

Overall, 25% (*n* = 99/400) of total samples were positive for *Acanthamoeba* spp. (18S rDNA gene) with a concentration range of 1.4 × 10^2^ to 2.33 × 10^6^ GU/L and 1.16 × 10^2^ to 3.63 × 10^8^ GU/swab (Supplementary Table S3). The prevalence of *Acanthamoeba* spp. was statistically significantly higher in residential samples than hospital samples (*p* = 0.001) (Table 1). There was also significantly higher prevalence in biofilm samples than water (*p* = 0.001), specifically residential biofilms (*p* = 0.001) (Table 1).

*Acanthamoeba* spp. was the only target OPPP whose presence was significantly positively correlated with all other target OPPPs in one or more sample sites. In residential water, *Acanthamoeba* spp. significantly positively correlated with *Legionella* spp. (*ρ* = 0.321, *p* = 0.013), *L. pneumophila* (*ρ* = 0.385, *p* = 0.003) and *V. vermiformis* (*ρ* = 0.333, *p* = 0.01) (Figure 1A), and in biofilm *Acanthamoeba* spp. was significantly positively correlated with *P. aeruginosa* (*ρ* = 0.706, *p* = 0.001) and *A. baumannii* (*ρ* = 0.535, *p* = 0.001) (Figure 1B). In hospital water, *Acanthamoeba* spp. was significantly positively correlated with *L. pneumophila* (*ρ* = 0.249, *p* = 0.002) and *S. aureus* (*ρ* = 0.336, *p* = 0.001) (Figure 2A), and in hospital biofilm *Acanthamoeba* spp. was significantly positively correlated with *Legionella* spp. (*ρ* = 0.236, *p* = 0.028) and *V. vermiformis* (*ρ* = 0.305, *p* = 0.004) (Figure 2B).

Within residential buildings, *Acanthamoeba* spp. prevalence was significantly higher in premises that had electric and solar hot water systems compared with gas (*p* = 0.001). However, there was no difference between buildings that did or did not have hot water storage. *Acanthamoeba* spp.

prevalence was positively correlated with plumbing system age (*ρ* = 0.308, *p* = 0.001). Prevalence also increased as outlet usage decreased in residential buildings (*ρ* = −0.251, *p* = 0.004), however there was no significant correlation between usage and prevalence in hospitals (*ρ* = −0.09, *p* = 0.211) (Figure 3).

### 3.3 Bacteria

#### 3.3.1 Pseudomonas aeruginosa

Overall, 41% (*n* = 123/299) of total samples were positive for *P. aeruginosa* (*gyrB* gene) with a concentration range of 1.08 × 10^3^ to 1.3 × 10^7^ GU/L and 1.36 × 10^2^ to 1.67 × 10^10^ GU/swab (Supplementary Table S3). The prevalence of *P. aeruginosa* was statistically significantly higher in residential samples compared with hospital samples (*p* = 0.001) (Table 1). There was also significantly higher prevalence in biofilm samples than water (*p* = 0.001) (Table 1). This was driven by the prevalence in residential biofilm samples, as *P. aeruginosa* prevalence was higher in hospital water than hospital biofilm (*p* = 0.035) (Table 1).

In residential water, *P. aeruginosa* prevalence was significantly negatively correlated with *Legionella* spp. (*ρ* = −0.37, *p* = 0.008) and *L. pneumophila* (*ρ* = −0.314, *p* = 0.025) (Figure 1A), and in biofilm *P. aeruginosa* prevalence was significantly positively correlated with *Acanthamoeba* spp. (*ρ* = 0.706, *p* = 0.001) and *A. baumannii* (*ρ* = 0.531, *p* = 0.001) (Figure 1B). Conversely, there were no significant relationships between *P. aeruginosa* and any other target OPPP in hospital water or biofilm (Figures 2A,B).

Within residential buildings, *P. aeruginosa* prevalence was significantly higher in buildings with electric hot water heaters compared to gas (*p* = 0.001). However, there was no significant difference between buildings that did or did not have hot water storage (*p* = 0.272). As plumbing system age increased, *P. aeruginosa* prevalence increased significantly (*ρ* = 0.344, *p* = 0.001), however, there was no significant correlation with building age (*ρ* = 0.109, *p* = 0.244). Prevalence also increased as outlet usage decreased in residential buildings (*ρ* = −0.245, *p* = 0.007) but not in hospitals (*ρ* = 0.05, *p* = 0.585) (Figure 3).

#### 3.3.2 Staphylococcus aureus

Overall, 26% (*n* = 71/273) of total samples were positive for *S. aureus* (*nuc* gene) with a concentration range of 4.73 × 10^3^ to 3.27 × 10^9^ and 1.5 × 10^2^ to 2.62 × 10^8^ GU/swab (Supplementary Table S3). There was no significant difference in *S. aureus* prevalence between residential or hospital samples (*p* = 0.084), or between water and biofilm samples (*p* = 0.634) (Table 1).

In residential water, *S. aureus* prevalence was significantly positively correlated with *Legionella* 16S (*ρ* = 0.293, *p* = 0.044) (Figure 1A), and in biofilms *S. aureus* prevalence significantly positively correlated with *L. pneumophila* (*ρ* = 0.268, *p* = 0.01) (Figure 1B). In hospital water, *S. aureus* prevalence was significantly positively correlated with *Acanthamoeba* spp. (*ρ* = 0.336, *p* = 0.001) (Figure 2A), however no significant correlations were seen in hospital biofilms (Figure 2B).

Unlike *P. aeruginosa* there was no significant difference in *S. aureus* prevalence between residential hot water systems (*p* = 0.919) or storage (*p* = 1). Similarly, there was no significant correlation between *S. aureus* prevalence and building age (*ρ* = −0.017, *p* = 0.859) or plumbing system age (*ρ* = −0.036, *p* = 0.702). In both residential and hospital samples, *S. aureus* prevalence increased as usage decreased ((*ρ* = −0.143, *p* = 0.125) and (*ρ* = −0.225, *p* = 0.027) respectively) (Figure 3).

#### 3.3.3 *Legionella* spp.

Overall, 26% (*n* = 104/400) of total samples were positive for *Legionella* spp. (16S rDNA gene) with a concentration range of 1 × 10^2^ to 2.8 × 10^6^ GU/L and 1.3 × 10^1^ to 7.7 × 10^4^ GU/swab (Supplementary Table S3). The prevalence of *Legionella* spp. was statistically significantly higher in residential samples than hospital samples (*p* = 0.001). There was also significantly higher prevalence in water than in biofilm (*p* = 0.001) (Table 1).

In residential water, *Legionella* spp. prevalence was significantly positively correlated with *L. pneumophila* (*ρ* = 0.803, *p* = 0.001), *Acanthamoeba* spp. (*ρ* = 0.321, *p* = 0.013), *V. vermiformis* (*ρ* = 0.328, *p* = 0.011), *S. aureus* (*ρ* = 0.293, *p* = 0.044) and significantly negatively correlated with *P. aeruginosa* (*ρ* = −0.37, *p* = 0.008) (Figure 1A), and in residential biofilms *Legionella* spp. prevalence was significantly positively correlated with *A. baumannii* (*ρ* = 0.288, *p* = 0.006) (Figure 1B). In hospital water, *Legionella* spp. prevalence was significantly positively correlated with *L.* *pneumophila* (*ρ* = 0.765, *p* = 0.001) and *V. vermiformis* (*ρ* = 0.518, *p* = 0.001) (Figure 2A), and in hospital biofilms *Legionella* spp. prevalence was significantly positively correlated with *L. pneumophila* (*ρ* = 0.567, *p* = 0.001) (Figure 2B).

Within residential buildings, *Legionella* spp. prevalence was significantly higher in properties with electric or gas hot water systems when compared with solar (*p* = 0.06 and 0.002 respectively). However, there was no significant difference between buildings that did or did not have hot water storage (*p* = 1). As plumbing system age decreased, there was a significant increase in *Legionella* spp. prevalence (*ρ* = −0.208, *p* = 0.01), however, there was no significant correlation with building age (*ρ* = 0.124, *p* = 0.126). *Legionella* spp. prevalence increased significantly as usage decreased in residential buildings (*ρ* = −0.301, *p* = 0.001) however, not in hospitals (*ρ* = −0.047, *p* = 0.517) (Figure 3).

#### 3.3.4 Legionella pneumophila

Overall, 24% (*n* = 95/400) of total samples were positive for *L. pneumophila* (*mip* gene) with a concentration range of 4 × 10^1^ to 3.5 × 10^5^ GU/L and 5 × 10^1^ to 1.12 × 10^6^ GU/swab (Supplementary Table S3). The prevalence of *L. pneumophila* was statistically significantly higher in residential samples than hospital samples (*p* = 0.019) (Table 1). There was also significantly higher prevalence in water than biofilm overall (*p* = 0.001), driven by the prevalence in hospital water (Table 1).

In residential water, *L. pneumophila* was significantly positively correlated with *Legionella* spp. (*ρ* = 0.803, *p* = 0.001), *Acanthamoeba* spp. (*ρ* = 0.385, *p* = 0.003), *V. vermiformis* (*ρ* = 0.316, *p* = 0.015) and significantly negatively correlated with *P. aeruginosa* (*ρ* = −0.314, *p* = 0.025) (Figure 1A), and in residential biofilm *L. pneumophila* was significantly positively correlated with *S. aureus* (*ρ* = 0.268, *p* = 0.01) (Figure 1B). In hospital water, *L. pneumophila* prevalence was significantly positively correlated with *Legionella* spp. (*ρ* = 0.765, *p* = 0.001), *Acanthamoeba* spp. (*ρ* = 0.249, *p* = 0.002) and *V. vermiformis* prevalence (*ρ* = 0.389, *p* = 0.001) (Figure 2A), and in hospital biofilm *L. pneumophila* prevalence was significantly positively correlated with *Legionella* spp. (*ρ* = 0.567, *p* = 0.001) and *V. vermiformis* (*ρ* = 0.319, *p* = 0.003) (Figure 2B).

In residential buildings, *L. pneumophila* prevalence was significantly higher in buildings with solar hot water heating systems compared to electric (*p* = 0.028). As both building age and plumbing system age decreased, *L. pneumophila* prevalence increased (*ρ* = −0.233, *p* = 0.008) and (*ρ* = −0.208, *p* = 0.019) respectively). In both residential and hospital buildings, there was no significant correlation between outlet usage and *L. pneumophila* prevalence ((*ρ* = 0.2, *p* = 0.818) and (*ρ* = −0.029, *p* = 0.682), respectively (Figure 3).

#### 3.3.5 Acinetobacter baumannii

Overall, 14% (*n* = 42/293) of total samples (residential and hospital) were positive for *A. baumannii* (*ompA* gene) with a concentration range of 2.67 × 10^2^ to 2.4 × 10^3^ GU/L and 1.36 × 10^2^ to 3.33 × 10^5^ GU/swab (Supplementary Table S3). The prevalence of *A. baumannii* was statistically significantly higher in residential samples compared to hospital samples (*p* = 0.001) (Table 1). Prevalence was also significantly higher in biofilm compared to water samples (*p* = 0.001) (Table 1).

In residential water, *A. baumannii* prevalence was not significantly correlated with any other target OPPP prevalence (Figure 1A), however in residential biofilms *A. baumannii* prevalence was significantly positively correlated with *Legionella* spp. (*ρ* = 0.288, *p* = 0.006), *Acanthamoeba* spp. (*ρ* = 0.535, *p* = 0.001) and *P. aeruginosa* prevalence (*ρ* = 0.531, *p* = 0.001) (Figure 1B). In both hospital water and biofilm, *A. baumannii* was not significantly correlated with any other target OPPP prevalence (Figures 2A,B).

Residential buildings with solar hot water heating systems had significantly higher *A. baumannii* prevalence compared with those with gas systems (*p* = 0.007), however, there was no difference between buildings with or without hot water storage (*p* = 1). *A. baumannii* prevalence increased significantly as residential building age decreased (*ρ* = −0.109, *p* = 0.032), however, there was no significant relationship with plumbing system age (*ρ* = −0.23, *p* = 0.792). *A. baumannii* was the only target pathogen increased in prevalence as outlet usage increased in residential buildings (*ρ* = 0.311, *p* = 0.001), however, there was no significant relationship between usage and prevalence in hospital buildings (*ρ* = 0.028, *p* = 0.766) (Figure 3).

## 4 Discussion

While previous research has studied the occurrence of individual opportunistic premise plumbing pathogens (OPPPs) such as *L. pneumophila*, the surveillance for multiple OPPPs and their protozoan hosts across different building types and outlets is still not well understood (Nisar et al., 2022; Isaac and Sherchan, 2020; Gomez-Alvarez et al., 2023; Shaheen et al., 2019; Lee-Masi et al., 2023; Waak et al., 2018). This study provides new quantitative information about the distribution of clinically relevant OPPPs across multiple settings. The high detection frequencies of these pathogens in both water and biofilm samples indicate their persistence and growth in drinking water plumbing systems, a niche thought to be a hostile environment for functionally complex microbiological communities (Jeanvoine et al., 2019).

### 4.1 Prevalence of opportunistic premise plumbing pathogens

The relationships between OPPPs in drinking water and biofilm samples in this study were found to be inconsistent, suggesting complex interactions between different microbial species. Notably, the presence of *L. pneumophila* negatively correlated with *P. aeruginosa* in residential water (*ρ* = −0.314, *p* = 0.025) and biofilm samples (*ρ* = −0.161, *p* = 0.126) (Figure 1), indicating that the presence of one pathogen may suppress the other under certain environmental conditions. Previous research has demonstrated that *P. aeruginosa* may antagonize *L. pneumophila* when in biofilms due to the production of bacteriocins or homoserine lactone quorum sensing (Abu Khweek and Amer, 2018; Mallegol et al., 2012). However, this inhibition deteriorates when *Klebsiella pneumonia* is also present in the biofilm (Stewart et al., 2012). Conversely, significant positive correlations were observed between *P. aeruginosa* and *A. baumannii* (*ρ* = 0.535, *p* = 0.001), and between *L. pneumophila* and *S. aureus* (*ρ* = 0.268, *p* = 0.01) in residential biofilm samples (Figure 1). Interestingly, these relationships were not seen in hospital biofilm samples. A broader perspective on antimicrobial AMR underscores the need to consider not only the prevalence of OPPPs but also the mechanisms by which these pathogens adapt and survive in water systems. The presence of AMR genes, particularly in biofilms, significantly influences pathogen persistence and resistance to common water treatment strategies. Carbapenem resistance genes have been shown to play a critical role in enhancing biofilm formation and strength for those made by both *P. aeruginosa* and *A. baumannii* (Azizi et al., 2015; Heydari and Eftekhar, 2015; Sherif et al., 2021). However, the extent to which the presence of these AMR genes contributes to the overall survival and virulence of multispecies biofilms in environments, like drinking water plumbing, is yet to be explored. Given the prevalence of AMR pathogens in biofilms, this highlights the need for updated water treatment guidelines that specifically target multi-species biofilm communities and incorporate antimicrobial resistance factors. Recent research has found a high prevalence of *Staphylococcus* spp. at the end points of DWDS and that they may remain dormant deep within biofilm matrices (Batista et al., 2022; Li et al., 2021). This physical protection from other OPPPs affords increased resistance to disinfection when compared to planktonic cells (Gholipour et al., 2024; Li et al., 2021). In the present study, *S. aureus* was found ubiquitously throughout both hospital and residential samples. Despite the growing body of evidence demonstrating that it is possible for *S. aureus* to contaminate drinking water plumbing systems and cause HAI outbreaks, it continues to be overlooked in drinking water treatment protocols (French et al., 2004; Ziwa et al., 2019; Sexton et al., 2011; Hayward et al., 2024). *Acanthamoeba* spp. was the only OPPP whose presence significantly positively correlated with all bacterial pathogens screened in this study. This finding reinforces the critical role that free-living amoebae play in biofilms, where they act as reservoirs for bacterial pathogens, complicating disinfection efforts and highlighting the need for targeted interventions to address these complex biofilm-host interactions (Nisar et al., 2022; Thomas and Ashbolt, 2011). This protection complicates water treatment efforts that are designed to target planktonic bacteria. The growth of pathogens in biofilms is shaped not only by the diversity and abundance of microorganisms, but also by the type of interactions between them. This finding underscores the potential risks of focusing treatment or disinfection strategies on a single species. Until these environments are recognized as a niche for diverse microbial communities, originating from both the incoming supply water and via end point contamination, treatment methods will remain ineffective.

### 4.2 Influence of building properties on opportunistic premise plumbing pathogens

One objective of this study was to investigate correlations between OPPP prevalence and abiotic factors such as building type, building and plumbing system age, outlet type and usage frequency. With healthcare at home increasingly promoted as a viable alternative to in-patient treatment, the residential drinking water plumbing environment must be acknowledged as a potential risk to patient health. *Acanthamoeba* spp., *P. aeruginosa, Legionella* spp., *L. pneumophila* and *A. baumannii* prevalence was significantly higher in residential samples compared to hospital samples (Table 1). Water utilities manage water treatment up to the property meter, but once water passes the meter, its quality becomes the responsibility of the property owner. While larger commercial buildings, including hospitals, often implement additional onsite water treatment to reduce the risk of waterborne healthcare-associated infections, this is rarely done in residential properties (Prest et al., 2016).

Prolonged water stagnation, often occurring in low-use fixtures or during periods of inactivity, creates conditions where disinfectant levels diminish, allowing OPPPs to thrive (Falkinham 3rd, 2015; Nisar et al., 2020; Lautenschlager et al., 2010). Regular flushing of outlets is recommended in healthcare premise plumbing management guidelines to mitigate this risk (World Health Organization, 2017; National Health and Medical Research Council, National Resource Management Ministerial Council, 2011). In the present study, *Acanthamoeba* spp., *V. vermiformis*, *P. aeruginosa* and *S. aureus* prevalence increased as outlet usage decreased (Figure 3). Conversely, *A. baumannii* prevalence increased as outlet usage increased (Figure 3). Considering *A. baumannii* prevalence was significantly higher in biofilm samples compared to water, this may be a result of end point contamination. As *A. baumannii* is an emerging AMR threat (Centers for Disease Control and Prevention, 2019a, World Health Organization, 2015), highlighting the need for updated strategies to manage this pathogen (Centers for Disease Control and Prevention, 2019a, World Health Organization, 2015). In addition to flushing, maintaining hot water storage above 60°C has been suggested as an accessible control mechanism for property owners. Electric and solar hot water heating systems strongly correlated with the prevalence of *Acanthamoeba* spp., *P. aeruginosa*, *L. pneumophila* and *A. baumannii* in the present study (Figure 3). Interestingly, there was no significant correlation between OPPP prevalence and if the residence had hot water storage or instantaneous water heating. The heating element in an electrically heated waterstorage tank is suspended in the water and does not come into contact with sediment at the bottom, which may harbor OPPPs (Bates et al., 2000; Martinelli et al., 2000). Instantaneous hot-water systems have been proposed as a better alternative to traditional continuous-flow or water-storage tanks, as they reduce the amount of warm water that remains stagnant in residential properties (Martinelli et al., 2000). Concerningly, 21% of sampled residents did not know what type of hot water system they had at their properties, and 30% did not know if they had hot water storage or an instantaneous system (Supplementary Table S3). Given that water temperature is considered one of the foundational barriers to control the growth and proliferation of these pathogens, this is an area that requires attention for future research and must be addressed when vulnerable individuals are receiving healthcare at home. Our findings emphasize the importance of routine maintenance and monitoring of water heating systems in residential and healthcare settings to prevent the growth of OPPPs.

Future research is needed to improve awareness of the role that residential water systems may play in the prevalence of OPPPS, particularly as vulnerable individuals receive healthcare at home.

## 5 Conclusion

Whilst previous research has studied the prevalence of individual opportunistic pathogens, there is limited knowledge understanding co-occurrence of multiple OPPPs and how building properties may influence their risk. Key findings include the prevalence of OPPPs in both water and biofilms from hospitals and residences, with *Acanthamoeba* showing a significant positive correlation with all bacterial OPPPs. Notably, a higher prevalence of OPPPs was found in biofilms compared to water, and residential properties exhibited a greater occurrence of these pathogens compared to hospitals. The public health relevance of these findings is significant, as they demonstrate that both hospital and residential drinking water systems can harbor a variety of opportunistic pathogens, which pose a potential risk for vulnerable populations, particularly those in healthcare settings. This study highlights how microbial drinking water quality can vary significantly between residential and hospital water systems, and how poor water system management may exacerbate OPPP persistence.

The high frequencies of detection of *Legionella* spp., *L. pneumophila*, *P. aeruginosa*, *S. aureus* and *A. baumannii* along with pathogenic protozoan hosts *Acanthamoeba* spp. and *V. vermiformis* particularly in residential properties, indicates their growth and persistence in treated drinking water plumbing systems. This research suggests that even in well treated drinking water systems, conditions may still permit the proliferation of diverse OPPPs, particularly in biofilms. Experimental validation of intervention strategies, such as flushing protocols and alternative disinfection methods, should be a focus of future research to reduce pathogen load. The growth of pathogens within biofilms was influenced not only by the diversity and abundance of microorganisms, but also by the nature of their interactions. For example, *P. aeruginosa* and *L. pneumophila* were significantly negatively correlated with one another in drinking water. This relationship has implications on water treatment approaches, as targeting one OPPP may inadvertently exacerbate the risk of another. Public health strategies should therefore focus on the complexities of biofilm-associated pathogens, their interactions, and their resistance to conventional disinfection methods. Current drinking water guidelines must recognize the increasing complexity of plumbing systems and the limitations of disinfection methods on dynamic bacterial communities. It is inadequate to depend solely on the water industry to ensure safe drinking water from treatment through to the point of use. Effective management requires collaborative efforts involving public health, water authorities, and healthcare institutions to address these emerging risks and to prioritize the development of new treatment technologies that target both planktonic and biofilm-associated pathogens.

## Data Availability

The raw data supporting the conclusions of this article will be made available by the authors, without undue reservation.
