# Supplementary material for: Microbial risks in drinking water systems: persistence and public health implications of opportunistic premise plumbing pathogens
Source: Front Microbiol. 2025 May 8;16:1575789. doi: 10.3389/fmicb.2025.1575789 (PMC12095195; doi:10.3389/fmicb.2025.1575789)
Supplement: Supplementary file 1 [file Data_Sheet_1.DOCX]

Supplementary Material

# Supplementary Data

Table S1- Sequences of oligos and fluorogenic probes, and reaction conditions used for qPCR assays

| Name | Sequence and Fluorescence signal (5’ – 3’) | Assay Conditions | Reference |
| --- | --- | --- | --- |
| *Acinetobacter baumannii* *ompA* gene | | | |
| Forward Primer | TCTTGGTGGTCACTTGAAGC | Hold: 95^o^C/5 min  45 cycles: 95^o^C/15 s, 60^o^C/30 s and 72^o^C/20 s  LOD: 29 GU/reaction | (McConnell Michael et al., 2012) |
| Reverse Primer | ACTCTTGTGGTTGTGGAGCA |  |  |
| Probe | 6FAM-AAGTTGCTCCAGTTGAACCAACTCCA- Iowa Black FQ |  |  |
| *Legionella pneumophila mip* gene | | | |
| Forward Primer | CCGATGCCACATCATTAGC | Hold: 95^o^C/5 min  45 cycles: 95^o^C/15 s, 60^o^C/30 s and 72^o^C/20 s  LOD: 35 GU/reaction | (International Organization for Standardization, 2019) |
| Reverse Primer | CCAATTGAGCGCCACTCATAG |  |  |
| Probe | 6FAM-TGCCTTTAGCCATTGCTTCCG-Iowa Black FQ |  |  |
| *Legionella* 16S rDNA gene | | | |
| Forward Primer | GGAGGGTTGATAGGTTAAGAGCT | Hold: 95^o^C/5 min  45 cycles: 95^o^C/15 s, 60^o^C/30 s and 72^o^C/20 s  LOD: 36.7 GU/reaction | (International Organization for Standardization, 2019) |
| Reverse Primer | CCAACAGCTAGTTGACATCGTTT |  |  |
| Probe | 6FAM-AGTGGCGAAGGCGGCTACCT- Iowa Black FQ |  |  |
| *Pseudomonas aeruginosa gyrB* gene | | | |
| Forward Primer | GGCGTGGGTGTGGAAGTC | Hold: 95^o^C/5 min  45 cycles: 95^o^C/15 s, 60^o^C/30 s and 72^o^C/20 s  LOD: 46.4 GU/reaction | (Lee et al., 2011) |
| Reverse Primer | TGGTGGCGATCTTGAACTTCTT |  |  |
| Probe | 6FAM-TGCAGTGGAACGACA- Iowa Black FQ |  |  |
| *Staphylococcus aureus nuc* gene | | | |
| Forward Primer | TGCCTTTACAGATAGCATGCCA | Hold: 95^o^C/5 min  45 cycles: 95^o^C/15 s, 60^o^C/30 s and 72^o^C/20 s  LOD: 44.4 GU/reaction | (Galia et al., 2019) |
| Reverse Primer | CCAGTAACGTCGAACGAATGAATGA |  |  |
| Probe | 6FAM-TCATTTCACGCAAACTGTTGGCC-Iowa Black FQ |  |  |
| *Acanthamoeba* 18s rDNA gene | | | |
| Forward Primer | CCCAGATCGTTT ACCGTGAA | Hold: 95^o^C/5 min  45 cycles: 95^o^C/15 s, 60^o^C/30 s and 72^o^C/20 s  LOD: 40.3 GU/reaction | (Qvarnstrom et al., 2006) |
| Reverse Primer | TAAATATTAATG CCCCCAACTATCC |  |  |
| Probe | CTGCCACCGAATACATT AGCATGG |  |  |
| *Vermamoeba vermiformia* 18S rDNA gene | | | |
| Forward Primer | TAACGATTGGAGGGCAAGTC | Hold: 95^o^C/5 min  45 cycles: 95^o^C/15 s, 60^o^C/30 s and 72^o^C/20 s  LOD: 44.3 GU/reaction | (Qvarnstrom et al., 2006) |
| Reverse Primer | ACGCCTGCTTTGAACACTCT |  |  |
| Probe | 6FAM-TGGGGAATCAACCGCTAGGA-Iowa Black FQ |  |  |

Table S2 – Residential sample abiotic factors

| **Sample ID** | **Building Type** | **Sample site** | **Sample type** | **Collector** | **Water heating System** | **Hot water storage** | **Building age (years)** | **Plumbing system age (years)** | **Usage** |
| --- | --- | --- | --- | --- | --- | --- | --- | --- | --- |
| R1 | House | Handbasin | Biofilm | CH | Did not know | Did not know | N/A | N/A | N/A |
| R10 | House | Handbasin | Biofilm | CH | Did not know | Did not know | N/A | N/A | N/A |
| R100 | House | Handbasin | Biofilm | CH | Electric | Did not know | More than 20 | More than 20 | 2 to 10/day |
| R101 | House | Handbasin | Biofilm | CH | Electric | Did not know | More than 20 | More than 20 | 2 to 10/day |
| R102 | Apartment | Handbasin | Biofilm | CH | Gas | Did not know | Less than 5 | Less than 5 | 1/day |
| R103 | Apartment | Handbasin | Biofilm | CH | Gas | Did not know | Less than 5 | Less than 5 | 1/day |
| R104 | House | Handbasin | Biofilm | CH | Electric | No | More than 20 | More than 20 | 2/day |
| R105 | House | Handbasin | Biofilm | CH | Electric | No | More than 20 | More than 20 | 2/day |
| R106 | House | Showerdrain | Biofilm | CH | Electric | Yes | More than 20 | More than 20 | 2 to 10/day |
| R107 | House | Showerhead | Biofilm | CH | Electric | Yes | More than 20 | More than 20 | 2 to 10/day |
| R108 | House | Bath Faucet | Biofilm | CH | Electric | No | More than 20 | More than 20 | Less than 1/month |
| R109 | House | Bath Drain | Biofilm | CH | Electric | No | More than 20 | More than 20 | Less than 1/month |
| R11 | House | Kitchen | Biofilm | CH | Did not know | Did not know | N/A | N/A | N/A |
| R12 | House | Showerhead | Biofilm | CH | Did not know | Did not know | N/A | N/A | N/A |
| R13 | House | Handbasin | Biofilm | CH | Did not know | Did not know | N/A | N/A | N/A |
| R14 | House | Kitchen | Biofilm | CH | Did not know | Did not know | N/A | N/A | N/A |
| R15 | House | Handbasin | Biofilm | CH | Did not know | Did not know | N/A | N/A | N/A |
| R16 | House | Handbasin | Biofilm | CH | Did not know | Did not know | N/A | N/A | N/A |
| R17 | House | Handbasin | Biofilm | CH | Did not know | Did not know | N/A | N/A | N/A |
| R18 | House | Handbasin | Biofilm | CH | Did not know | Did not know | More than 20 | N/A | More than 10/day |
| R19 | University housing | Handbasin | Biofilm | CH | Did not know | Did not know | More than 20 | N/A | More than 10/day |
| R2 | House | Kitchen | Biofilm | CH | Did not know | Did not know | N/A | N/A | N/A |
| R20 | House | Handbasin | Biofilm | CH | Electric | No | More than 20 | More than 20 | More than 10/day |
| R21 | House | Handbasin | Biofilm | CH | Electric | No | More than 20 | More than 20 | More than 10/day |
| R22 | House | Showerhead | Biofilm | CH | Did not know | Did not know | More than 20 | 10 to 14 | 2/day |
| R23 | House | Handbasin | Biofilm | CH | Did not know | Did not know | More than 20 | More than 20 | 2 to 10/day |
| R24 | House | Showerhead | Biofilm | CH | Gas | Did not know | N/A | N/A | 2 to 10/day |
| R25 | House | Showerdrain | Biofilm | CH | Gas | Did not know | N/A | N/A | 2 to 10/day |
| R26 | House | Handbasin | Biofilm | CH | Gas | No | More than 20 | More than 20 | 2 to 10/day |
| R27 | House | Handbasin | Biofilm | CH | Gas | No | More than 20 | More than 20 | 2 to 10/day |
| R28 | House | Handbasin | Biofilm | CH | Gas | No | More than 20 | Less than 5 | More than 10/day |
| R29 | House | Handbasin | Biofilm | CH | Gas | No | More than 20 | Less than 5 | More than 10/day |
| R3 | House | Kitchen | Biofilm | CH | Did not know | Did not know | N/A | N/A | N/A |
| R30 | N/A | Handbasin | Biofilm | CH | Did not know | Did not know | Less than 5 | Less than 5 | More than 10/day |
| R31 | N/A | Handbasin | Biofilm | CH | Did not know | Did not know | Less than 5 | Less than 5 | More than 10/day |
| R32 | House | Showerhead | Biofilm | CH | Electric | Did not know | More than 20 | More than 20 | 2 to 10/day |
| R34 | Apartment | Handbasin | Biofilm | CH | Did not know | Did not know | More than 20 | 10 to 14 | 2 to 10/day |
| R35 | Apartment | Handbasin | Biofilm | CH | Did not know | Did not know | More than 20 | 10 to 14 | 2 to 10/day |
| R36 | Apartment | Handbasin | Biofilm | CH | Gas | Did not know | Less than 5 | Less than 5 | 2 to 10/day |
| R37 | Apartment | Handbasin | Biofilm | CH | Gas | Did not know | Less than 5 | Less than 5 | 2 to 10/day |
| R38 | N/A | Kitchen | Biofilm | CH | Gas | Did not know | More than 20 | Less than 5 | More than 10/day |
| R39 | N/A | Kitchen | Biofilm | CH | Gas | No | More than 20 | Less than 5 | More than 10/day |
| R4 | House | Showerhead | Biofilm | CH | Did not know | Did not know | N/A | N/A | N/A |
| R40 | N/A | Handbasin | Biofilm | CH | Did not know | Did not know | 5 to 9 | 5 to 9 | 2 to 10/day |
| R41 | N/A | Handbasin | Biofilm | CH | Did not know | Did not know | 5 to 9 | 5 to 9 | 2 to 10/day |
| R44 | House | Kitchen | Biofilm | CH | Gas | Yes | More than 20 | 10 to 14 | 2 to 10/day |
| R45 | House | Kitchen | Biofilm | CH | Gas | Yes | More than 20 | 10 to 14 | 2 to 10/day |
| R46 | House | Handbasin | Biofilm | CH | Gas | No | Less than 5 | Less than 5 | 2 to 10/day |
| R47 | House | Handbasin | Biofilm | CH | Gas | No | Less than 5 | Less than 5 | 2 to 10/day |
| R5 | House | Handbasin | Biofilm | CH | Did not know | Did not know | N/A | N/A | N/A |
| R50 | House | Handbasin | Biofilm | CH | Gas | Yes | 5 to 9 | 5 to 9 | 2 to 10/day |
| R51 | House | Kitchen | Biofilm | CH | Gas | Yes | 5 to 9 | 5 to 9 | 2 to 10/day |
| R6 | House | Handbasin | Biofilm | CH | Did not know | Did not know | N/A | N/A | N/A |
| R60 | House | Handbasin | Biofilm | CH | Gas | Did not know | N/A | N/A | More than 10/day |
| R61 | House | Handbasin | Biofilm | CH | Gas | Did not know | N/A | N/A | More than 10/day |
| R68 | House | Kitchen | Biofilm | CH | Gas | No | Less than 5 | Less than 5 | 2 to 10/day |
| R69 | House | Kitchen | Biofilm | CH | Gas | No | Less than 5 | Less than 5 | 2 to 10/day |
| R7 | House | Handbasin | Biofilm | CH | Did not know | Did not know | N/A | N/A | N/A |
| R70 | House | Handbasin | Biofilm | CH | Gas | No | Less than 5 | Less than 5 | 2 to 10/day |
| R71 | House | Handbasin | Biofilm | CH | Gas | No | Less than 5 | Less than 5 | 2 to 10/day |
| R72 | House | Handbasin | Biofilm | CH | Gas | No | Less than 5 | Less than 5 | 2 to 10/day |
| R73 | House | Handbasin | Biofilm | CH | Gas | No | Less than 5 | Less than 5 | 2 to 10/day |
| R74 | N/A | Handbasin | Biofilm | CH | Did not know | Did not know | More than 20 | 10 to 14 | More than 10/day |
| R75 | N/A | Handbasin | Biofilm | CH | Did not know | Did not know | More than 20 | 10 to 14 | More than 10/day |
| R76 | House | Handbasin | Biofilm | CH | Electric | Yes | More than 20 | More than 20 | 2 to 10/day |
| R77 | House | Handbasin | Biofilm | CH | Electric | Yes | More than 20 | More than 20 | 2 to 10/day |
| R78 | House | Showerhead | Biofilm | CH | Gas | No | More than 20 | More than 20 | 2/day |
| R79 | House | Shower drain | Biofilm | CH | Gas | No | More than 20 | More than 20 | 2/day |
| R8 | House | Handbasin | Biofilm | CH | Did not know | Did not know | N/A | N/A | N/A |
| R80 | House | Handbasin | Biofilm | CH | Electric | Yes | More than 20 | More than 20 | More than 10/day |
| R81 | House | Handbasin | Biofilm | CH | Electric | Yes | More than 20 | More than 20 | More than 10/day |
| R84 | House | Handbasin | Biofilm | CH | Electric | No | More than 20 | More than 20 | 2/day |
| R85 | House | Handbasin | Biofilm | CH | Electric | No | More than 20 | More than 20 | 2/day |
| R86 | House | Handbasin | Biofilm | CH | Electric | No | More than 20 | More than 20 | 2/day |
| R87 | House | Handbasin | Biofilm | CH | Electric | No | More than 20 | More than 20 | 2/day |
| R88 | House | Bath Drain | Biofilm | CH | Electric | Yes | More than 20 | More than 20 | 1/fortnight |
| R89 | House | Bath Faucet | Biofilm | CH | Electric | Yes | More than 20 | More than 20 | 1/fortnight |
| R9 | House | Handbasin | Biofilm | CH | Did not know | Did not know | N/A | N/A | N/A |
| R90 | House | Bath Drain | Biofilm | CH | Electric | Did not know | More than 20 | More than 20 | 2 to 10/day |
| R91 | House | Bath Faucet | Biofilm | CH | Electric | Did not know | More than 20 | More than 20 | 2 to 10/day |
| R94 | House | Showerhead | Biofilm | CH | Electric | Yes | More than 20 | More than 20 | 2 to 10/day |
| R95 | House | Showerdrain | Biofilm | CH | Electric | Yes | More than 20 | More than 20 | 2 to 10/day |
| R96 | House | Bath Faucet | Biofilm | CH | Electric | Yes | More than 20 | More than 20 | 1/day |
| R97 | House | Bath Drain | Biofilm | CH | Electric | Yes | More than 20 | More than 20 | 1/day |
| R98 | House | Handbasin | Biofilm | CH | Electric | Yes | More than 20 | More than 20 | More than 10/day |
| R99 | House | Handbasin | Biofilm | CH | Electric | Yes | More than 20 | More than 20 | More than 10/day |
| 58S1 | N/A | Shower | Water | MAN | Gas | No | More than 20 | 5 to 9 | 2 to 10/day |
| 58S2 | N/A | Shower | Water | MAN | Gas | No | More than 20 | 5 to 9 | 2 to 10/day |
| 60T | N/A | Tap faucet | Biofilm | MAN | Gas | No | More than 20 | 5 to 9 | More than 10/day |
| 68S1 | N/A | Shower | Water | MAN | Gas | No | More than 20 | 5 to 9 | 2 to 10/day |
| 68S2 | N/A | Shower | Water | MAN | Gas | No | More than 20 | 5 to 9 | 2 to 10/day |
| 71S1 | N/A | Shower | Water | MAN | Gas | No | More than 20 | 5 to 9 | 2 to 10/day |
| 71S2 | N/A | Shower | Water | MAN | Gas | No | More than 20 | 5 to 9 | 2 to 10/day |
| 71T1 | N/A | Tap faucet | Biofilm | MAN | Gas | No | More than 20 | 5 to 9 | More than 10/day |
| 71T2 | N/A | Tap faucet | Biofilm | MAN | Gas | No | More than 20 | 5 to 9 | More than 10/day |
| 74S1 | N/A | Shower | Water | MAN | Gas | No | More than 20 | 5 to 9 | 2 to 10/day |
| 74S2 | N/A | Shower | Water | MAN | Gas | No | More than 20 | 5 to 9 | 2 to 10/day |
| 74T | N/A | Tap faucet | Biofilm | MAN | Gas | No | More than 20 | 5 to 9 | More than 10/day |
| 75S1 | N/A | Shower | Water | MAN | Gas | No | More than 20 | 5 to 9 | 2 to 10/day |
| 75S2 | N/A | Shower | Water | MAN | Gas | No | More than 20 | 5 to 9 | 2 to 10/day |
| AE01 | N/A | Shower | Water | MAN | Electric | No | N/A | Less than 5 | 2 to 10/day |
| AWDS | N/A | Shower | Water | MAN | Did not know | Did not know | N/A | N/A | N/A |
| AWUS | N/A | Shower | Water | MAN | Did not know | Did not know | N/A | N/A | N/A |
| CH01 | House | Shower | Water | MAN | Gas | No | More than 20 | More than 20 | 2/day |
| CH02 | House | Shower | Water | MAN | Gas | No | More than 20 | More than 20 | 1/day |
| CT01 | N/A | Shower | Water | MAN | Did not know | Did not know | N/A | N/A | N/A |
| EF01 | N/A | Shower | Water | MAN | Gas | No | More than 20 | Less than 5 | 2/day |
| EK01 | N/A | Shower | Water | MAN | Gas | No | More than 20 | N/A | 2 to 10/day |
| EK03 | N/A | Shower | Water | MAN | Gas | No | Less than 5 | Less than 5 | 2/day |
| EK04 | N/A | Shower | Water | MAN | Gas | No | Less than 5 | Less than 5 | Less than 1/month |
| FL2-1 | Apartment | Shower | Water | MAN | Gas | No | More than 20 | Less than 5 | 2 to 10/day |
| FL2-2 | Apartment | Shower | Water | MAN | Gas | No | More than 20 | Less than 5 | 2 to 10/day |
| FL2-3 | Apartment | Shower | Water | MAN | Gas | No | More than 20 | Less than 5 | 2 to 10/day |
| FL2-4 | Apartment | Shower | Water | MAN | Gas | No | More than 20 | Less than 5 | 2 to 10/day |
| FL2-5 | Apartment | Shower | Water | MAN | Gas | No | More than 20 | Less than 5 | 2 to 10/day |
| FL2-6 | Apartment | Shower | Water | MAN | Gas | No | More than 20 | Less than 5 | 2 to 10/day |
| FL3-1 | Apartment | Shower | Water | MAN | Gas | No | More than 20 | 5 to 9 | 2 to 10/day |
| FL3-2 | Apartment | Shower | Water | MAN | Gas | No | More than 20 | 5 to 9 | 2 to 10/day |
| FL3-3 | Apartment | Shower | Water | MAN | Ga | No | More than 20 | 5 to 9 | 2 to 10/day |
| FL3-4 | Apartment | Shower | Water | MAN | Gas | No | More than 20 | 5 to 9 | 2 to 10/day |
| HW01 | House | Shower | Water | MAN | Gas | No | More than 20 | Less than 5 | 2/day |
| HW02 | House | Shower | Water | MAN | Gas | No | More than 20 | Less than 5 | 1/week |
| HW03 | House | Shower | Water | MAN | Gas | No | Less than 5 | Less than 5 | Less than 1/month |
| HW04 | House | Shower | Water | MAN | Gas | No | Less than 5 | Less than 5 | Less than 1/month |
| HW05 | House | Shower | Water | MAN | Gas | No | Less than 5 | Less than 5 | 2/day |
| JW01 | House | Shower | Water | MAN | Gas | No | More than 20 | 5 to 9 | 1/week |
| JW02 | House | Shower | Water | MAN | Gas | No | More than 20 | 5 to 9 | 2/day |
| KRDS | House | Shower | Water | MAN | Electric | Yes | More than 20 | 10 to 14 | 1/week |
| KRUS | House | Shower | Water | MAN | Electric | Yes | More than 20 | 10 to 14 | 1/day |
| KS01 | N/A | Shower | Water | MAN | Gas | No | More than 20 | Less than 5 | 1/day |
| KS02 | N/A | Shower | Water | MAN | Gas | No | More than 20 | Less than 5 | 1/day |
| KS03 | N/A | Shower | Water | MAN | Gas | No | More than 20 | Less than 5 | 1/day |
| KS04 | N/A | Shower | Water | MAN | Gas | No | More than 20 | Less than 5 | Less than 1/month |
| MW01 | N/A | Shower | Water | MAN | Solar | Yes | More than 20 | Less than 5 | 2/day |
| NK01 | N/A | Shower | Water | MAN | Solar | Yes | More than 20 | Less than 5 | 2/day |
| NM01 | N/A | Shower | Water | MAN | Did not know | Did not know | More than 20 | More than 20 | 2 to 10/day |
| NS01 | N/A | Shower | Water | MAN | Electric | Yes | N/A | N/A | 1/day |
| P1 | N/A | Tap fauc | Biofilm | MAN | Gas | No | More than 20 | 10 to 14 | N/A |
| P2 | N/A | Tap fauc | Biofilm | MAN | Gas | No | More than 20 | 10 to 14 | N/A |
| P3 | N/A | Tap fauc | Biofilm | MAN | Gas | No | More than 20 | 10 to 14 | N/A |
| P4 | N/A | Tap fauc | Biofilm | MAN | Gas | No | More than 20 | 10 to 14 | N/A |
| PT01 | N/A | Shower | Water | MAN | Gas | No | More than 20 | More than 20 | 1/day |
| PT04 | N/A | Shower | Water | MAN | Electric | Yes | More than 20 | Less than 5 | 1/day |
| PT05 | N/A | Shower | Water | MAN | Electric | Yes | More than 20 | Less than 5 | Less than 1/month |
| PT06 | N/A | Shower | Water | MAN | Electric | Yes | More than 20 | Less than 5 | 1/day |
| RJ01 | N/A | Shower | Water | MAN | Gas | No | 5 to 9 | 5 to 9 | 2 to 10/day |
| RJ02 | N/A | Shower | Water | MAN | Gas | No | 5 to 9 | 5 to 9 | Less than 1/month |
| SB01 | N/A | Shower | Water | MAN | Electric | Yes | 5 to 9 | 5 to 9 | 1/day |
| SB02 | N/A | Shower | Water | MAN | Electric | Yes | 5 to 9 | 5 to 9 | 1/day |
| SB03 | N/A | Shower | Water | MAN | Electric | Yes | 5 to 9 | 5 to 9 | 1/day |
| T0 | N/A | Tap faucet | Biofilm | MAN | Gas | No | More than 20 | 10 to 14 | More than 10/day |
| TD01 | N/A | Shower | Water | MAN | Gas | No | More than 20 | 10 to 14 | 2 to 10/day |
| TD02 | N/A | Shower | Water | MAN | Gas | No | More than 20 | 10 to 14 | 1/day |
| TK01 | N/A | Shower | Water | MAN | Gas | Did not know | N/A | Less than 5 | 2 to 10/day |

N/A = Data not collected

Table S3 – Hospital abiotic factors

| **Sample ID** | **Sample Site** | **Sample Type** | **Collector** | **One month prior to sampling (flow count)** |
| --- | --- | --- | --- | --- |
| 1 | Basin | Biofilm | CH | 125 |
| 2 | Basin | Biofilm | CH | 88 |
| 3 | Basin | Biofilm | CH | 163 |
| 4 | Basin | Biofilm | CH | 147 |
| 5 | Basin | Biofilm | CH | 206 |
| 6 | Shower | Biofilm | CH | 55 |
| 7 | Basin | Biofilm | CH | 88 |
| 8 | Shower | Biofilm | CH | 152 |
| 9 | Basin | Biofilm | CH | 104 |
| 10 | Shower | Biofilm | CH | 7 |
| 11 | Shower | Biofilm | CH | 242 |
| 12 | Basin | Biofilm | CH | 161 |
| 13 | Shower | Biofilm | CH | 37 |
| 14 | Basin | Biofilm | CH | 79 |
| 15 | Shower | Biofilm | CH | 39 |
| 16 | Basin | Biofilm | CH | 44 |
| 17 | Basin | Biofilm | CH | 27 |
| 18 | Shower | Biofilm | CH | 247 |
| 19 | Basin | Biofilm | CH | 359 |
| 20 | Shower | Biofilm | CH | 183 |
| 21 | Basin | Biofilm | CH | 235 |
| 22 | Basin | Biofilm | CH | 422 |
| 23 | Basin | Water | CH | 163 |
| 24 | Basin | Water | CH | 35 |
| 25 | Basin | Water | CH | 125 |
| 26 | Shower | Water | CH | 125 |
| 27 | Basin | Water | CH | 183 |
| 28 | Basin | Water | CH | 147 |
| 29 | Shower | Water | CH | 242 |
| 30 | Shower | Water | CH | 383 |
| 31 | Basin | Water | CH | 88 |
| 32 | Basin | Water | CH | 44 |
| 33 | Shower | Water | CH | 55 |
| 34 | Basin | Water | CH | 206 |
| 35 | Shower | Water | CH | 152 |
| 36 | Basin | Water | CH | 62 |
| 37 | Shower | Water | CH | 202 |
| 38 | Basin | Water | CH | 235 |
| 39 | Shower | Water | CH | 7 |
| 40 | Basin | Water | CH | 79 |
| 41 | Shower | Water | CH | 37 |
| 42 | Basin | Water | CH | 27 |
| 43 | WC | Water | CH | 267 |
| 44 | WC | Water | CH | N/A |
| 45 | Shower | Water | CH | 39 |
| 46 | Basin | Water | CH | 104 |
| 47 | Shower | Water | CH | 422 |
| 48 | Shower | Water | CH | 242 |
| 49 | Basin | Water | CH | 247 |
| 50 | WC | Water | CH | 64359 |
| 51 | Basin | Water | CH | 161 |
| 52 | Shower | Water | CH | 359 |
| 61 | Basin | Water | CH | N/A |
| 62 | Basin | Water | CH | N/A |
| 63 | Basin | Water | CH | N/A |
| 65 | Shower | Water | CH | N/A |
| 66 | Basin | Water | CH | N/A |
| 67 | Basin | Water | CH | N/A |
| 68 | Basin | Water | CH | N/A |
| 70 | Basin | Water | CH | N/A |
| 71 | Basin | Water | CH | N/A |
| 72 | Tap | Biofilm | CH | N/A |
| 73 | Drain | Biofilm | CH | N/A |
| 74 | Tap | Biofilm | CH | N/A |
| 75 | Faucet | Biofilm | CH | N/A |
| 76 | Drain | Biofilm | CH | N/A |
| 77 | Tap | Biofilm | CH | N/A |
| 78 | Faucet | Biofilm | CH | N/A |
| 79 | Drain | Biofilm | CH | N/A |
| 80 | Tap | Biofilm | CH | N/A |
| 81 | Faucet | Biofilm | CH | N/A |
| 82 | Drain | Biofilm | CH | N/A |
| 83 | Faucet | Biofilm | CH | N/A |
| 84 | Drain | Biofilm | CH | N/A |
| 85 | Tap | Biofilm | CH | N/A |
| 86 | Faucet | Biofilm | CH | N/A |
| 87 | Drain | Biofilm | CH | N/A |
| 88 | Tap | Biofilm | CH | N/A |
| 89 | Faucet | Biofilm | CH | N/A |
| 90 | Drain | Biofilm | CH | N/A |
| 10B | Faucet | Biofilm | MAN | N/A |
| 11B | Faucet | Biofilm | MAN | N/A |
| 12B | TMV | Biofilm | MAN | N/A |
| 13B | TMV | Biofilm | MAN | N/A |
| 14B | TMV | Biofilm | MAN | 280 |
| 1B | Faucet | Biofilm | MAN | 2 |
| 2B | Faucet | Biofilm | MAN | 2 |
| 3B | Faucet | Biofilm | MAN | 2 |
| 4B | TMV | Biofilm | MAN | 109 |
| 5B | TMV | Biofilm | MAN | 109 |
| 6B | Faucet | Biofilm | MAN | 96 |
| 7B | Faucet | Biofilm | MAN | 96 |
| 8B | TMV | Biofilm | MAN | N/A |
| 9B | TMV | Biofilm | MAN | N/A |
| B63R20 | Basin | Water | MAN | 94 |
| HBR12T76 | Basin | Water | MAN | 421 |
| HBR12T76 | Basin | Water | MAN | 477 |
| HBR12T76 | Basin | Water | MAN | 531 |
| R13B | Basin | Water | MAN | 175 |
| R13B | Basin | Water | MAN | 46 |
| R13B | Basin | Water | MAN | 123 |
| R13S | Shower | Water | MAN | 437 |
| R15B | Basin | Water | MAN | 72 |
| R15B | Basin | Water | MAN | 477 |
| R15B | Basin | Water | MAN | 207 |
| R15B | Basin | Water | MAN | 221 |
| R15S | Shower | Water | MAN | 330 |
| R15S | Shower | Water | MAN | 418 |
| R15S | Shower | Water | MAN | 416 |
| R15S | Shower | Water | MAN | 796 |
| R17B | Basin | Water | MAN | 148 |
| R17S | Shower | Water | MAN | 796 |
| R19B | Basin | Water | MAN | 307 |
| R19B | Basin | Water | MAN | 253 |
| R19B | Basin | Water | MAN | 4 |
| R19B | Basin | Water | MAN | 109 |
| R19S | Shower | Water | MAN | 210 |
| R19S | Shower | Water | MAN | 325 |
| R19S | Shower | Water | MAN | 59 |
| R19S | Shower | Water | MAN | 141 |
| R20B | Basin | Water | MAN | 325 |
| R20B | Basin | Water | MAN | 273 |
| R20B | Basin | Water | MAN | 192 |
| R20S | Shower | Water | MAN | 18 |
| R20S | Shower | Water | MAN | 64 |
| R20S | Shower | Water | MAN | 57 |
| R20S | Shower | Water | MAN | 54 |
| R21B | Basin | Water | MAN | N/A |
| R21B | Basin | Water | MAN | 102 |
| R21B | Basin | Water | MAN | 357 |
| R21B | Basin | Water | MAN | N/A |
| R21S | Shower | Water | MAN | 2 |
| R21S | Shower | Water | MAN | 313 |
| R21S | Shower | Water | MAN | 10 |
| R21S | Shower | Water | MAN | 136 |
| R22B | Basin | Water | MAN | 267 |
| R22B | Basin | Water | MAN | 188 |
| R22B | Basin | Water | MAN | 152 |
| R22B | Basin | Water | MAN | 363 |
| R22S | Shower | Water | MAN | 192 |
| R22S | Shower | Water | MAN | 251 |
| R22S | Shower | Water | MAN | 135 |
| R22S | Shower | Water | MAN | 324 |
| R23B | Basin | Water | MAN | 206 |
| R23S | Shower | Water | MAN | N/A |
| R24B | Basin | Water | MAN | 290 |
| R24B | Basin | Water | MAN | 358 |
| R24B | Basin | Water | MAN | 71 |
| R24B | Basin | Water | MAN | 131 |
| R24S | Shower | Water | MAN | 429 |
| R24S | Shower | Water | MAN | 390 |
| R24S | Shower | Water | MAN | 163 |
| R24S | Shower | Water | MAN | 147 |
| R25B | Basin | Water | MAN | 883 |
| R25B | Basin | Water | MAN | 355 |
| R25B | Basin | Water | MAN | 176 |
| R25B | Basin | Water | MAN | 242 |
| R25S | Shower | Water | MAN | 889 |
| R25S | Shower | Water | MAN | 168 |
| R25S | Shower | Water | MAN | 85 |
| R25S | Shower | Water | MAN | 361 |
| R27B | Basin | Water | MAN | N/A |
| R27S | Shower | Water | MAN | N/A |
| R28S | Shower | Water | MAN | N/A |
| R29B | Basin | Water | MAN | 148 |
| R29B | Basin | Water | MAN | 171 |
| R29B | Basin | Water | MAN | 288 |
| R29B | Basin | Water | MAN | 257 |
| R29S | Shower | Water | MAN | 423 |
| R29S | Shower | Water | MAN | 437 |
| R29S | Shower | Water | MAN | 413 |
| R29S | Shower | Water | MAN | 127 |
| R31B | Basin | Water | MAN | 269 |
| R31B | Basin | Water | MAN | 286 |
| R31B | Basin | Water | MAN | 538 |
| R31B | Basin | Water | MAN | 139 |
| R31S | Shower | Water | MAN | 304 |
| R31S | Shower | Water | MAN | 462 |
| R31S | Shower | Water | MAN | 244 |
| R31S | Shower | Water | MAN | 359 |
| R32B | Basin | Water | MAN | 217 |
| R32B | Basin | Water | MAN | 258 |
| R32B | Basin | Water | MAN | 400 |
| R32B0T1 | Basin | Water | MAN | 96 |
| R32B0T2 | Basin | Water | MAN | 96 |
| R32S | Shower | Water | MAN | 102 |
| R32S | Shower | Water | MAN | 144 |
| R32S | Shower | Water | MAN | 148 |
| R32S0T1 | Shower | Water | MAN | 109 |
| R32S0T2 | Shower | Water | MAN | 109 |
| R33B | Basin | Water | MAN | 414 |
| R33B | Basin | Water | MAN | 229 |
| R33B | Basin | Water | MAN | 46 |
| R33B | Basin | Water | MAN | 334 |
| R33S | Shower | Water | MAN | N/A |
| R33S | Shower | Water | MAN | 295 |
| R33S | Shower | Water | MAN | 361 |
| R33S | Shower | Water | MAN | N/A |
| R34B | Basin | Water | MAN | N/A |
| R34B | Basin | Water | MAN | N/A |
| R34B | Basin | Water | MAN | 59 |
| R34S | Shower | Water | MAN | 132 |
| R34S | Shower | Water | MAN | 103 |
| R34S | Shower | Water | MAN | 363 |
| S1 | Shower | Biofilm | MAN | 388 |
| S10 | Shower | Biofilm | MAN | 363 |
| S2 | Shower | Biofilm | MAN | 388 |
| S22 | TMV | Biofilm | MAN | 530 |
| S23 | Shower | Biofilm | MAN | 210 |
| S24 | Basin | Biofilm | MAN | 307 |
| S25 | TMV | Biofilm | MAN | 54 |
| S26 | Basin | Biofilm | MAN | 139 |
| S27 | Shower | Biofilm | MAN | 390 |
| S28 | TMV | Biofilm | MAN | 147 |
| S29 | Basin | Biofilm | MAN | 123 |
| S3 | Faucet | Biofilm | MAN | 123 |
| S30 | Basin | Biofilm | MAN | 206 |
| S31 | TMV | Biofilm | MAN | 530 |
| S32 | Basin | Biofilm | MAN | 363 |
| S33 | TMV | Biofilm | MAN | 54 |
| S34 | Shower | Biofilm | MAN | 144 |
| S35 | Shower | Biofilm | MAN | 359 |
| S36 | TMV | Biofilm | MAN | 147 |
| S37 | Faucet | Biofilm | MAN | 450 |
| S38 | Basin | Biofilm | MAN | 147 |
| S4 | Shower | Biofilm | MAN | 64 |
| S5 | Shower | Biofilm | MAN | 64 |
| S6 | Faucet | Biofilm | MAN | 400 |
| S7 | Shower | Biofilm | MAN | 148 |
| S8 | Faucet | Biofilm | MAN | 59 |
| S9 | Shower | Biofilm | MAN | 363 |
| STB15 | Basin | Water | MAN | N/A |
| STB15 | Basin | Water | MAN | 530 |
| STB15 | Basin | Water | MAN | N/A |
| VWCT69 | Basin | Water | MAN | 147 |
| VWCT69 | Basin | Water | MAN | 269 |
| VWCT69 | Basin | Water | MAN | 192 |
| VWCT69 | Basin | Water | MAN | 416 |
| W1 | Basin | Water | MAN | N/A |
| W11 | Basin | Biofilm | MAN | 450 |
| W12 | Basin | Biofilm | MAN | 450 |
| W13 | Basin | Biofilm | MAN | 520 |
| W14 | Basin | Biofilm | MAN | 520 |
| W15 | Basin | Biofilm | MAN | 147 |
| W2 | Basin | Water | MAN | N/A |
| W3 | Basin | Water | MAN | N/A |

N/A = data not collected

Table S3 – Minimum and maximum microbial concentrations present in the positive water and biofilm samples

| Target pathogen | Minimum Concentration | Maximum concentration |
| --- | --- | --- |
| **Water (GU/L)** | | |
| *Vermamoeba vermiformis* | 2.7 x 10^2^ | 7.47 x 10^7^ |
| *Acanthamoeba* spp. | 1.40 x 10^2^ | 2.33 x 10^6^ |
| *Pseudomonas aeruginosa* | 1.08 x 10^3^ | 1.3 x 10^7^ |
| *Staphylococcus aureus* | 4.73 x 10^3^ | 3.27 x 10^9^ |
| *Legionella* spp. | 1 x 10^2^ | 2.8 x 10^6^ |
| *Legionella pneumophila* | 4.0 x 10^1^ | 3.5 x 10^5^ |
| *Acinetobacter baumannii* | 2.67 x 10^2^ | 2.4 x 10^3^ |
| **Biofilm (GU/swab)** | | |
| *Vermamoeba vermiformis* | 1.2 x 10^2^ | 3.45 x 10^8^ |
| *Acanthamoeba* spp. | 1.16 x 10^2^ | 3.63 x 10^8^ |
| *Pseudomonas aeruginosa* | 1.36 x 10^3^ | 1.67 x 10^10^ |
| *Staphylococcus aureus* | 1.5 x 10^2^ | 2.62 x 10^8^ |
| *Legionella* spp. | 1.3 x 10^1^ | 7.7 x 10^4^ |
| *Legionella pneumophila* | 5.0 x 10^1^ | 1.12 x 10^6^ |
| *Acinetobacter baumannii* | 1.36 x 10^2^ | 3.33 x 10^5^ |
